# Crossing two sperm chromatin-localized mCherry transgenes into a single *C. elegans* strain boosts signal intensity without harming sperm function

**DOI:** 10.17912/micropub.biology.000214

**Published:** 2020-02-12

**Authors:** Amanda C Wong, Jiajia He, Ashley R Wiltsie, Victoria S Krawiec, Gillian M Stanfield

**Affiliations:** 1 Department of Human Genetics, University of Utah, Salt Lake City, UT 84112

**Figure 1:**
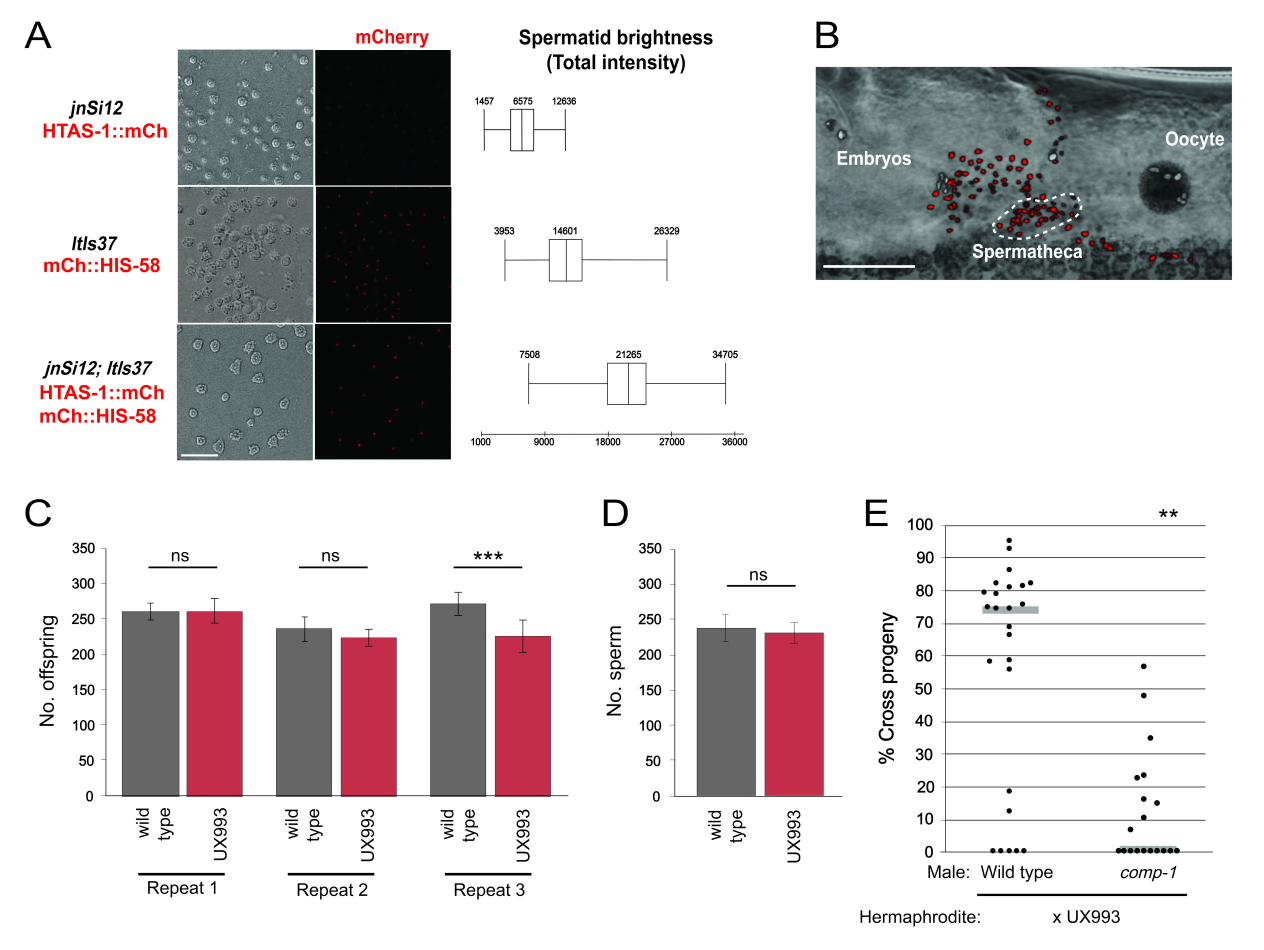
Signal intensity and sperm parameters in the UX993 strain. (A) Images and brightness of spermatid nuclei from UX993 and its parent strains. (B) Single perspective of a 3-D rendering generated from a z stack taken through the gonad. The brightfield image is overlaid with fluorescence. When imaged *in vivo,* UX993 sperm nuclei (red) are clearly visible, and the sperm populations in the oviduct, spermatheca, and uterus are easily distinguishable from one another. Female germline and oocyte nuclei are pseudocolored white. The cluster of sperm within the spermatheca is indicated by the white dotted line. (C) UX993 hermaphrodite fertility is comparable to that of wild-type hermaphrodites. Bar graphs show average progeny counts excluding those from the first 24 hours post-L4; error bars indicate 95% confidence intervals. ***, p<0.01; ns, not significant (Student’s t test). (D) N2 and UX993 hermaphrodites have indistinguishable numbers of sperm. Bar graph shows average sperm counts in 24-hour post-L4 hermaphrodites; error bars indicate 95% confidence intervals. n=15-16. (E) UX993 hermaphrodite sperm have wild-type usage patterns: they are outcompeted by wild-type male sperm, and they outcompete competition-deficient *comp-1* male sperm. Each dot represents one mating, and results for one representative repeat are shown. Gray lines indicate median values. **, p<0.001 (Kolmogorov-Smirnov test). Scale bars, 20 µm.

## Description

A formidable barrier to imaging studies of *in vivo* *C. elegans* sperm behavior has been the lack of bright, sperm-specific fluorescent markers. Use of dim sperm markers negatively influences the ability to perform confocal imaging in various ways, including but not limited to loss of signal deep into the gonad, which can be ~40 mm thick (Hubbard and Greenstein 2000); loss of spatial and temporal resolution, due to techniques commonly used to address imaging dim signals, such as signal accumulation; and increased phototoxicity and bleaching, due to increased excitation intensity. For *in vivo* imaging of the *C. elegans* hermaphrodite gonad, temporal resolution is limited by the rapidity of sheath contraction (McCarter *et al.* 1999) and the small physical size of sperm cells, parameters that already push the limits of many imaging systems. Researchers may find themselves trapped in the so-called “pyramid of frustration,” which refers to the difficult position of balancing the conflicting demands of high contrast, spatial resolution, temporal resolution, and sample health (Laissue *et al.* 2017).

Various existing techniques used for sperm imaging exist, each with unique strengths and weaknesses. For example, vital dyes such as Mitotracker Red CMXRos and Nile Blue A have been used to track sperm guidance and motility within the uterus (Hu *et al.* 2019) and to transiently follow sperm movement in the spermatheca (Ward and Carrel 1979). SYTO dyes have also been used to visualize sperm nucleic acids (Singson *et al.* 1999; Hill and L’Hernault 2001). However, none of these dyes are sperm-specific, and some photobleach rapidly. Dyes also can be lost from sperm due to diffusion. Nile Blue A, in particular, moves from male sperm to the hermaphrodite’s spermathecal walls within minutes of spermathecal entry, prohibiting observation of sperm movements soon after entry (Ward and Carrel 1979). Alternatively, strains with germ line-specific expression have been generated using bombardment to integrate low copy-number transgenes (Praitis *et al.* 2001), with MosSCI (Mos1-mediated Single Copy Insertion), which inserts single transgene copies into defined sites (Frøkjaer-Jensen *et al.* 2008), and with CRISPR/Cas9-based knock-in strategies. Elements of the inserted transgene’s structure, such as promoter choice, presence of introns, and placement of the fluorescent protein relative to fusion partner(s), have been documented to increase fluorophore expression level and intensity (Okkema *et al.* 1993; Zeiser *et al.* 2011; Takayama and Onami 2016; Nance and Frøkjaer-Jensen 2019). However, creating transgenic strains requires specialized knowledge of cloning and microinjection, and requires weeks to months to achieve.

Here, we describe a strain with enhanced sperm nuclear fluorescence, which we generated by crossing *jnSi12* and *ltIs37*, two unlinked sperm histone::mCherry transgenes, into the same strain. The transgene *jnSi12* expresses a variant histone HTAS-1::mCherry fusion protein specifically in sperm (Chu *et al.* 2006, GM Stanfield and AK Snow, unpublished). The transgene *ltIs37* expresses an mCherry::HIS-58 fusion protein throughout the germ line (McNally *et al.* 2006). The new strain, UX993, is homozygous for both markers and has mCherry expression in all germline nuclei. Nuclei of oocytes and the more proximal germ line are visually distinct from those of sperm, and can provide useful orientation information. The above strategy is advantageous because it can be executed by anyone with knowledge of basic worm husbandry in a relatively short time frame. We also describe assays for comparing the brightness of different strains and for testing that strains retain wild-type fertility and sperm competitive advantage.

Both *in vivo* and *in vitro*, UX993 sperm nuclei were brighter than those of its parent strains. UX993 spermatid nuclei exhibited higher total intensities when observed *in vitro* (Fig. 1A). UX993 hermaphrodites have brood sizes that are frequently indistinguishable from those of N2 wild-type hermaphrodites (Fig. 1C). As in the N2 strain, sperm counts are comparable to progeny counts, suggesting every sperm fertilizes an oocyte (Ward and Carrel 1979)(Fig. 1D). Importantly, UX993 hermaphrodites show wild-type usage in crosses to males. They are strongly outcompeted for fertilization by wild-type male sperm, such that matings nearly always result in a majority of cross progeny (Fig. 1E). In addition, they have improved success when competing with *comp-1* male sperm, such that matings yield a decreased percentage of cross progeny (Hansen *et al.* 2015)(Fig. 1E). Taken together, our results indicate that UX993 has functionally wild-type mCherry-marked sperm that are significantly brighter than those of its parents, and it is suitable for studying the cellular dynamics of sperm competition using *in vivo* imaging.

We report a strain (UX993) in which two existing markers (*jnSi12* and *ltIs37*) for the same sperm structure (nuclei/chromatin) have been combined, leading to a significant improvement in fluorescent brightness compared to the parent strains (UX972 and OD56), without harming sperm function. We have validated UX993 for use in *in vivo*microscopy studies of *C. elegans* reproductive cell biology. The ability to image other sperm structures also might be improved by a similar strategy. Here we used pre-existing, sperm-specific markers, which may not always be readily available. Thus, it remains worthwhile to generate additional markers, using MosSCI, CRISPR/Cas9, or other methods yet to be developed. Nevertheless, UX993 should be a useful tool to tighten the corners of the pyramid of frustration for sperm imaging experiments.

## Methods

**Analysis of Spermatid Nuclear Fluorescence Intensity** For *in vitro* imaging, UX993, UX972, and OD56 males were isolated from hermaphrodites as mid-L4 stage larvae and their spermatids were examined at approximately 29 hours post-L4 (Shakes and Ward 1989). Briefly, the virgin males were dissected in Sperm Medium containing 10 mM dextrose, 1 mM MgSO4, 5 mM CaCl2, 50 mM NaCl, 25 mM KCl, and 5.5 mM HEPES pH 7.8. Slides were coverslipped and sealed with Vaseline, and spermatids were imaged with a Leica TCS SP8 resonant scanning confocal microscope using the settings detailed in the table below.

**Table:** 

**Parameter**	**Setting**
Dimensions (x, y)	512 x 512
Fluorescent laser excitation	mCherry: 594 nm, 10% intensity
Step size	0.43 um
Objective	HC PL APO CS2 40X/1.10 WATER
Zoom	2
Line accumulation	8
Pinhole	1 AU
Detectors	mCherry: HyD, gain 20% Brightfield: PMT, gain 308
White light laser intensity	70%

Spermatid nuclei were manually segmented using Fluorender (Wan *et al* 2009). For each spermatid, contiguous pixels in the fluorescent channel that coincide with nuclei seen in the brightfield channel were selected. The voxel count (sumN) and mean voxel intensity of each selection were calculated using the Component Analyzer function. The sumN and average voxel intensity values were multiplied to generate a total intensity value for each spermatid nucleus. Sperm that were obscured or whose nuclei were not fully in the field of view were excluded from analysis. Between 775-850 spermatids from 5-7 animals per genotype were analyzed.

For *in vivo* imaging, UX993, OD56, and UX972 hermaphrodites at 24 hours post-L4 were mounted in a polyethylene glycol hydrogel, as described (Burnett *et al.* 2018), and imaged on a Leica SP8 using parameters similar to those used for *in vitro* spermatids, with the exception of dimensions (488 x 250) and zoom (2.75).

**Sperm Competition and Fertility Assays** Sperm competition assays were performed as described by Hansen *et al.* (2015). Briefly, UX707 or UX708 mid-L4 males and UX993 mid-L4 hermaphrodites were placed in 1:1 ratios on freshly seeded NGM plates. After 48 hours, both parents were removed. Adult offspring were scored as either cross progeny (GFP-positive pharynx) or self progeny (GFP-negative pharynx), and the percentage of cross progeny generated by each mating was calculated. Three experimental repeats were performed (n=14-25 per genotype). Figure 1E shows representative results.

To compare UX993 hermaphrodite fertility to that of wild-type N2, mid-L4 hermaphrodites were individually placed on NGM plates and moved to a fresh plate every 24 hours until egg laying ceased. Progeny were counted after all had reached at least the L4 stage. Three repeats of this experiment were performed. For direct comparison with the sperm count data, data in Fig. 1C exclude progeny produced in the first 24 hours post-L4. From 13-20 hermaphrodites of each genotype were examined in each experimental repeat.

**Sperm Counts** Staged 24 hour post mid-L4 UX993 or N2 hermaphrodites were fixed using Carnoy’s fixative, stained with Vectashield Mounting Medium containing DAPI (Vector Laboratories, Inc, Burlingame, CA), and imaged using a 40X/1.30NA EC Plan-Neofluar oil objective with an AxioImager M1 compound microscope (Zeiss). Sperm nuclei were manually counted using the ImageJ Point Tool function (Schneider *et al.* 2012). Gonad arms in which all sperm nuclei could not clearly be seen were excluded from analysis. A total of 15 N2 and 16 UX993 hermaphrodites were examined.

## Reagents

**Strains and Husbandry** *C. elegans* nematodes were grown at 20°C on NGM (nematode growth medium) plates seeded with the *Escherichia coli* strain OP50 (Brenner 1974). The strains used were: UX993 *jnSi12[peel-1p::htas-1::mCh::3’tbb-2, Cb-unc-119(+)]II; ezIs2[fkh-6::GFP,unc-119(+)]III; ltIs37[(pAA64)pie-1p::mCh::his-58, unc-119(+)]IV*, UX972 *jnSi12[Ppeel-1::htas::mCh:3’tbb-2, Cb-unc-119(+)] II; ezIs2[fkh-6:GFP, unc-119(+)] III*, OD56 *unc-119(ed3)III; ltIs37[(pAA64)pie-1p::mCh::his-58, unc-119(+)]IV*, Bristol N2, UX707 *mIs11[myo-2::GFP, pes-10::GFP, gut::GFP] IV; him-5(ok1896) V*, and UX708 *comp-1(gk1149) I; mIs11[myo-2::GFP, pes-10::GFP, gut::GFP] IV; him-5(ok1896) V*. UX993 has been deposited at the Caenorhabditis Genetics Center.
